# Medicine for the soul: (Non)religious identity, coping, and mental health during the COVID-19 pandemic

**DOI:** 10.1371/journal.pone.0296436

**Published:** 2024-01-02

**Authors:** Claire Peneycad, Renate Ysseldyk, Emily Tippins, Hymie Anisman

**Affiliations:** 1 Department of Health Sciences, Carleton University, Ottawa, Canada; 2 Department of Neuroscience, Carleton University, Ottawa, Canada; University Kebangsaan Malaysia, MALAYSIA

## Abstract

Although the threat and uncertainty of the COVID-19 pandemic has become a significant source of distress, using religion to cope may be associated with more positive health. Given the severity and chronicity of the pandemic, religious individuals may also have relied on a variety of *non*-religious coping methods. Much of the existing COVID-19 research overlooks the role of religious group membership and beliefs in relation to coping responses and associated mental health, with an additional lack of such research within the Canadian context. Thus, this cross-sectional study investigated relations among religiosity, stressor appraisals, (both religious and non-religious) coping strategies, mental and physical health in a religiously-diverse Canadian community sample (*N* = 280) during the pandemic’s 2^nd^ wave from March to June 2021. Numerous differences were apparent in appraisal-coping methods and health across five (non)religious groups (i.e., Atheists, Agnostics, “Spiritual but not religious”, Christians, and those considered to be religious “Minorities” in Canada). Religiosity was also associated with better mental health, appraisals of the pandemic as a challenge from which one might learn or grow, and a greater reliance on problem-focused, emotional-engagement, and religious coping. Moreover, both problem-focused and emotional-engagement coping mediated the relations between religiosity and health. Taken together, this research has implications for individual-level coping as well as informing culturally-sensitive public health messages promoting targeted self-care recommendations with integrated religious or spiritual elements during times of threat and uncertainty, such as the COVID-19 pandemic.

## Introduction

Since the onset of the COVID-19 pandemic in March 2020, the SARS-CoV-2 virus has had lasting impacts. COVID-19 has taken a toll on the physical health of individuals around the world, while governments and health care systems have been overwhelmed, and almost 7 million people had died as of October, 2023 [1]. In addition to the physical health disturbances posed by COVID-19, the pandemic resulted in harmful repercussions to mental health [2]. The health and fatality risks of COVID-19, heightened distress related to risk of infection, as well as isolation due to government-imposed social distancing and lockdown measures have resulted in poor mental health outcomes with lasting impacts world-wide [2–4].

The pandemic evoked a wide range of coping methods to deal with the ongoing threat [5, 6]. As with responses to a variety of stressors, some of these coping strategies have been more adaptive (e.g., support-seeking, problem-solving) than have others (e.g., drug and alcohol use, rumination) [7]. Moreover, the use of diverse stressor appraisal and coping strategies may vary depending on an array of socio-demographic factors, group memberships, and identities [8–10]. In the context of the present investigation, stressor appraisals refer to an individual’s evaluation of a stressful event, whereas coping strategies refer to an individual’s cognitions or behaviours used to manage the stress [11]. This study investigated associations among self-reported religious beliefs and identities, stressor appraisals, coping strategies, as well as mental and physical health during the COVID-19 pandemic in a diverse community sample of participants across Canada. In view of the effectiveness of religiosity as a coping strategy among some individuals [12–15], it was anticipated that those who identified with a religious group (compared to those who did not) would self-report more adaptive stressor appraisals, coping, and health outcomes. Given associations among various appraisals and coping strategies with health [16–18], it was also expected that appraisal-coping processes would mediate the relations between religiosity and self-reported health.

### COVID-19 and mental health

The COVID-19 health threat has become a significant and chronic source of distress associated with increased mental health symptoms, including depression and anxiety. A nationally-representative study of Canadians revealed that symptoms of anxiety were reported to be four times higher during—compared to before—the COVID-19 pandemic, and reports of depressive symptoms nearly doubled [2]. Similarly, distress levels among Americans early in the COVID-19 pandemic were notably high, as were symptoms of depression and anxiety [3]. Individuals considered to be at higher risk of severe COVID-19 infections, including older adults and those with underlying medical issues, were found to be at an even greater risk of experiencing anxiety symptoms [19]. Comparable outcomes had similarly been reported during the SARS epidemic from 2002–2004, where social disengagement, stress, and anxiety resulted in higher rates of suicide deaths among older adults [20].

Many countries implemented lockdowns and restrictions of varying degrees in an attempt to prevent further viral spread of COVID-19 and relieve overwhelmed health care systems. These restrictions were helpful in reducing the number of COVID-19 cases and in protecting physical health [21], but lockdowns may have also contributed to poor mental health outcomes. Indeed, the traumatic stress responses of quarantined or isolated parents and children during an earlier pandemic revealed that 25% of parents and 30% of children met the criteria for post-traumatic stress disorder (PTSD) [22]. More recent studies likewise indicated elevated psychological distress in populations that experienced COVID-19-related quarantine measures, including increased prevalence of panic disorder, anxiety, and depression [4]. As COVID-19 continues to affect the lives of people around the world, along with the potential for similar events occurring in the future, it is imperative that the mental health implications of such scenarios are better understood so that prophylactic measures can be supported when the next pandemic emerges.

### Religion, coping, and mental health

Considerable research has indicated that relying on religion to cope with stress is often associated with more positive mental health outcomes [12, 14, 15, 23–26]. Indeed, various functions of religion might provide possible explanations for improved mental health, such as providing meaning in life, strong community networks and social support, and having ideologies that promote healthy lifestyle behaviours [27]. For example, the association between greater belief in God and having a greater sense of meaning in life has been associated with reduced levels of depression [28]. In addition, having a greater sense of meaning in life may also offer hope to individuals experiencing adversity and may thereby facilitate coping with stressful events and traumatic experiences [29]. From a social perspective, the importance of community—and associated collective group identity—inherent to many religions can also provide individuals with strong social support networks when faced with stressful situations [30–34]. Finally, many religions promote healthy practices and thought processes as part of their ideologies that often increase self-control [35], mindfulness, and gratitude, each of which have been linked to increased positive mental health outcomes [25, 36].

Beyond these positive lifestyle choices and ideologies, the strong influence of religious beliefs and spirituality on coping strategies—including an array of non-religious strategies—have been associated with mental health outcomes [37–41]. Specifically, such studies have provided evidence that religious identity and beliefs are often linked to more adaptive stressor appraisals and coping strategies. In the field of religion and coping, religious coping is considered as the translation of common religious beliefs into specific ways to cope with stress [42, 43], notwithstanding that religious coping itself can take both positive (e.g., prayer [44]) and negative (e.g., anger at God, [45]) forms with each shown to be habitually associated with either positive or negative mental health outcomes, respectively [12, 15, 46, 47]. Whereas religious coping requires previously established religious beliefs, the use of non-religious coping methods (e.g., rumination, social support seeking, humour) to cope with stressors may or may not be accompanied by such beliefs [42, 43]. As such, religious individuals also engage in a variety of appraisals and coping strategies that are *not* directly related to their religious beliefs or identity. For example, religious individuals may be more likely to appraise stressful events as challenging rather than threatening [48, 49]. Moreover, religious individuals may engage in both religious and non-religious social support-seeking as an emotional-engagement coping strategy, as well as endorse problem-focused coping strategies to cope with adverse events [33, 41].

Within the context of the COVID-19 pandemic, it has been reported that Google searches using the word “prayer” reached a record high in March 2020, the timing of which corresponds with the onset of the pandemic [50, 51]. Consistent with the notion that people often rely on their religious beliefs to cope with traumatic events, interest in religion surged throughout the course of the pandemic [50, 52]. However, many pandemic-related restriction measures, such as stay-at-home orders and indoor capacity limits, might have been a source of distress for religious individuals in particular [26, 51, 53]. In particular, many religions value routine religious social gatherings and worship services, and previous research suggests a positive association between religious service attendance and positive mental health [24, 26, 54]. Lockdowns and restrictions greatly limited these opportunities, possibly contributing to poorer mental health outcomes among religious individuals [5, 54]. Indeed, in the absence of regular contact with one’s religious group members during the pandemic, religious individuals may have relied more heavily on a variety of non-religious appraisals and coping strategies. However, to our knowledge, no research has examined the use of non-religious appraisal-coping processes among religious (compared to non-religious) individuals in the COVID-19 pandemic context.

### The present research

Although considerable research has been conducted on mental health outcomes associated with various life stressors, there has been less focus on the role of religious group membership and beliefs in relation to coping responses and associated mental health, specifically within the context of COVID-19. Moreover, much of this research has been conducted in non-Canadian contexts [3, 6, 13, 19, 23, 51], where social and political factors (including national pandemic responses) may differentially affect outcomes. For example, the Canadian health care system provides publicly-funded, universal access to medical services, whereas many other countries do not (most notably the USA, where much of the existing research has been conducted). In addition, such social and political factors, including political leadership, public opinion and compliance, economic considerations, and vaccine rollout were unique to each country’s response to the COVID-19 pandemic. This, in turn, affected the total number of COVID-19 cases, deaths, as well as vaccine doses administered. At the beginning of our data collection (March 15^th^, 2021), there were approximately 900,000 cumulative confirmed COVID-19 cases and 22,000 cumulative confirmed COVID-19 deaths in Canada, with 7% of the Canadian population having received at least one dose of the COVID-19 vaccine [55]. Thus, the present study examined religious group identity and beliefs, stressor appraisals, (both religious and non-religious) coping strategies, as well as mental and physical health in a multi-cultural Canadian sample during the COVID-19 pandemic. Potential differences in appraisal-coping responses, mental, and physical health were examined across a variety of (non)religious groups. In addition, relations among religiosity, appraisals, coping, and health, as well as the potential mediating roles of appraisal-coping processes in the links between religiosity and health, were examined in this pandemic context.

## Method

### Participants and procedure

A total of 392 people participated in an online study through Amazon Mechanical Turk (MTurk)–a crowdsourcing website (http://www.mturk.com) and were compensated $3.00 CAD. After giving written informed consent, participants completed questionnaires assessing religiosity, stressor appraisals, coping strategies, and general mental and physical health. The study was available to people over 18 years of age living in Canada and IP addresses were collected to determine geo-location. Responses included 142 from Ontario (Canada’s most heavily populated province), 34 from Quebec, 32 from British Columbia, 24 from Alberta, 10 from Nova Scotia, 7 from New Brunswick, 6 from Manitoba, 4 from Saskatchewan, and 1 from Newfoundland and Labrador.

Following the removal of 112 incomplete or invalid survey responses, the final sample totalled 280 participants (142 men, 137 women, 1 other; ranging in age from 18–72 years, *M* = 37.35, *SD* = 11.19). This sample size provided 95% power to detect medium effect sizes (two-tailed; [56]), based on our primary analyses (ANOVA and MANOVA) and the most conservative estimate is reported for conciseness. Data was collected between March 15^th^ and June 4^th^, 2021, one full year into the COVID-19 pandemic. Approval from the Carleton University Research Ethics Board-B (CUREB-B) was obtained prior to the commencement of data collection.

A demographic questionnaire was also completed by participants, in which they self-identified their age, gender, ethnic/racial identity, religious affiliation, highest degree or level of education, and average annual household income. Participants were then categorized into a (non)religious group for our primary analyses of interest, with the remaining demographics treated as covariates. This categorization resulted in five (non)religious groups, namely Atheists (*n* = 69), Agnostics (*n* = 45), “Spiritual but not religious” (*n* = 53), Christians (*n* = 69), and those who identified as belonging to a variety of religious groups that are considered to be “Minorities” in Canada (i.e., Baháʼí, Buddhist, Hindu, Jewish, Muslim, and Sikh; *n* = 44).

### Measures

#### Religiosity

The Centrality of Religiosity Scale (CRS) [57] consisted of 13 questions to measure the general intensities of five theoretically defined dimensions of religiosity. Each item evaluated the frequency or intensity of the activation of religious constructs, rated on a scale ranging from 1 (*never/not at all*) to 5 (*very often/very much*). These dimensions included *intellectual* (e.g., “How often do you think about religious issues?”; 3 items; α = .86); *ideology* (e.g., “To what extent do you believe in the existence of God or something divine?”; 3 items; α = .93); *public practice* (e.g., “How often do you take part in religious services?”; 3 items; α = .93); *private practice* (e.g., “How often do you pray?” 2 items; α = .82); and *religious experience* (e.g., “How often do you experience situations in which you have the feeling that God or something divine intervenes in your life?”; 2 items; α = .94). A total religiosity score based on these five dimensions was also computed (α = .91).

#### Stressor appraisals

Stressor appraisals in the context of the COVID-19 pandemic were evaluated on seven dimensions [58], each consisting of four items rated on a scale ranging from 1 (*not at all*) to 5 (*extremely*). These dimensions included *threat appraisals* (e.g., “How threatening is this situation?”; 4 items; α = .75; *challenge appraisals* (e.g., “Is this going to have a positive impact on me?”; 4 items; α = .65); *centrality appraisals* (e.g., “Does this situation have important consequences for me?”; 4 items; α = .88); appraisals of the situation as *controllable-by-self* (e.g., “Do I have what it takes to do well in this situation?”; 4 items; α = .86); *controllable-by-others* (e.g., “Is there help available to me for dealing with this problem?”; 4 items; α = .85); and *uncontrollable by anyone* (e.g., “Is this a totally hopeless situation?”; 4 items; α = .80). The final dimension reflected the general *stressfulness* of the pandemic (e.g., “To what extent do I perceive this situation as stressful?”; 4 items; α = .78).

#### Coping

Coping with the COVID-19 pandemic was assessed with the short-version Survey of Coping Profile Endorsements [59], comprising 30-items, reflecting 15 distinct coping strategies (e.g. cognitive restructuring, rumination, social-support seeking, denial, etc.). Participants rated each item on a scale ranging from 0 (*not at all*) to 4 (*totally*). To reduce the number of coping dimensions a principal components analysis was conducted, which resulted in four factors. These factors included *emotional-engagement coping* (e.g., “I told others that I was really upset”; α = .81); *emotional-avoidance* (e.g., “I avoided thinking about the problem”; α = .66); *problem-focused coping* (e.g., “I made plans to overcome my concerns or the problem”; α = .70); and *religious coping* (e.g., “I turned to God or my faith”; single item).

#### Mental & physical health

Mental health was assessed with a widely used, global single-item measure [60], which asked participants to rate their current mental health ranging from 1 (*poor*) to 5 (*excellent*). To include a more comprehensive view of health, physical health was also assessed with a single-item measure [61], which asked participants to rate their current physical health ranging from 1 (*poor*) to 5 (*excellent*).

### Statistical analyses

A series of analyses of variance (ANOVAs) and multivariate analyses of variances (MANOVAs) were conducted to examine potential differences in appraisals, coping, mental and physical health, and religiosity among the five (non)religious groups. Analyses assessing (non)religious group differences and relations among stressor appraisals, coping, mental and physical health, and religiosity that included age, gender, race, education, and income as covariates yielded identical patterns of significant results. However, given that some participants chose not to report all demographics, to maximize sample size, the analyses presented here are those that include (non)religious group as the sole predictor.

Correlations then assessed relations among the variables of interest, and mediation analyses were conducted to determine whether appraisal-coping processes accounted for the relations between religiosity and health (i.e., mental and physical). To assess these multiple-mediation models, we conducted regression-based bootstrapping analyses with 95% confidence intervals (CIs) using Hayes’ (2022) PROCESS macro (Model 4) [62]; this analysis provides evidence of mediation if the significant direct effect between the predictor and outcome variable is reduced when the mediator(s) is included, and the 95% CI does not include zero.

## Results

### Demographics

Participants in the present study were diverse in terms of gender, age, education, income, ethnic, racial, and (non)religious identity. Table 1 presents demographics of this sample as a function of self-identified (non)religious group membership as well as total numbers across all (non)religious categories.

**Table 1:** Demographics of (non)religious groups.

	Atheist	Agnostic	Spiritual	Christian	Religious “Minority”	Totals
	(*n* = 69)	(*n* = 45)	(*n* = 53)	(*n* = 69)	(*n* = 44)	
**Gender**						
Women	26	20	33	23	35	137
Men	43	25	19	46	9	142
Other	0	0	1	0		1
**Age**						
18–29	15	19	8	18	11	60
30–49	47	24	41	32	27	171
50–64	7	2	4	13	6	32
65+	0	0	0	6	0	6
**Education**						
High school diploma or less	10	11	9	10	5	45
Bachelor’s degree	30	20	30	38	23	141
Master’s degree	12	7	4	7	14	44
PhD or higher	5	1	0	1	0	7
Trade certificate/diploma	11	6	10	13	2	42
**Household Income**						
Less than $25,000	2	3	5	9	6	25
$25,000 - $50,000	12	10	17	12	8	59
$50,000 - $100,000	29	18	22	24	21	114
$100,000 - $200,000	19	10	5	16	7	57
More than $200,000	0	0	2	6	0	8
Prefer not to say	7	4	2	2	2	17
**Ethnic/Racial Identity**						
Asian (i.e. Chinese, Japanese, Korean)	11	11	11	4	3	40
South Asian (i.e. East Indian, Pakistani, Punjabi, Sri Lankan)	1	0	2	1	25	29
South East Asian (i.e. Cambodian, Indonesian, Laotian)	1	4	1	3	4	13
Arabic	6	0	0	1	3	10
Black	0	1	2	3	0	6
South/Latin American	1	1	1	4	0	7
Mexican	0	0	0	0	0	0
Aboriginal	0	0	1	3	0	4
White	49	29	36	52	12	178

### Religious group differences in stressor appraisals

A MANOVA assessed whether differences in stressor appraisals (threat, challenge, centrality, controllable-by-self, controllable-by-others, uncontrollable, and general stressfulness) associated with the COVID-19 pandemic varied as a function of (non)religious group self-identification. This analysis revealed a significant multivariate effect, Pillai’s = 0.206; *F*(28,1088) = 2.11, *p* < .001, *η*^2^ = .052, resulting from differences across groups in appraisals of the pandemic as a challenge, *F*(4,275) = 4.44, *p* = .002, *η*^2^ = .061, as uncontrollable, *F*(4,275) = 3.23, *p* = .002, *η*^2^ = .059, and as controllable by others, *F*(4,275) = 2.96, *p* = .020, *η*^2^ = .041.

More specifically, as seen in Table 2, Atheists were significantly *less* likely to view the pandemic as a challenge from which they might grow compared to religious “Minorities” (*p* = .003) and Christians (*p* = .018). However, religious “Minorities” were more likely to report viewing the pandemic as uncontrollable compared to Christians (*p* = .055) and Agnostics (*p* = .002). Finally, Agnostics reported appraisals of the pandemic as controllable-by-others marginally (but not significantly) more than did those who were “Spiritual but not religious” (*p* = .094). There were no statistically significant differences across (non)religious groups in appraisals of the pandemic as central, threatening, controllable-by-self, or as generally stressful.

**Table 2:** Stressor appraisals (Means, SDs) as a function of (non)religious group.

	Atheist	Agnostic	Spiritual	Christian	Religious “Minority”	*F* (4,275)	*η* ^2^
Appraisals	(*n* = 69)	(*n* = 45)	(*n* = 53)	(*n* = 69)	(*n* = 44)		
Threat	2.85_a_ (0.88)	3.01_a_ (0.71)	3.17_a_ (0.89)	2.85_a_ (0.82)	3.19_a_ (0.77)	2.28	.032
Challenge	2.39_a_ (0.77)	2.54_a,b_ (0.76)	2.70_a,b_ (0.66)	2.80_b_ (0.81)	2.93_b_ (0.80)	4.44^**^	.061
Centrality	2.97_a_ (0.94)	3.39_a_ (0.82)	3.34_a_ (0.99)	3.24_a_ (0.87)	3.38_a_ (0.86)	2.30	.032
Controllable-by-self	3.37_a_ (0.98)	3.48_a_ (0.75)	3.33_a_ (0.85)	3.66_a_ (0.74)	3.34_a_ (0.78)	1.56	.022
Controllable-by-others	2.97_a_ (0.94)	3.39_a_ (0.78)	2.92_a_ (0.98)	3.31_a_ (0.80)	3.15_a_ (0.85)	2.96^*^	.041
Uncontrollable	2.30_a,b_ (0.85)	1.97_a_ (0.73)	2.47_a,b_ (0.76)	2.19_a_ (0.93)	2.66_b_ (1.02)	4.33^**^	.059
Stressfulness	2.86_a_ (0.89)	2.97_a_ (0.71)	3.20_a_ (0.83)	2.87_a_ (0.92)	3.18_a_ (0.68)	2.16	.030

^**^p < .01

^*^ *p* < .05; Means in the same row that do not share subscripts differ at *p* < .05 (with the exception of the difference in controllable-by-others appraisals between religious “Minorities” and Christians; *p* = .055).

### Religious group differences in coping

A MANOVA was conducted to assess whether differences in coping strategies (emotional-engagement, emotional-avoidance, problem-focused, and religious coping) used to manage stress associated with the COVID-19 pandemic varied as a function of (non)religious group identity. The analysis revealed a significant multivariate effect, Pillai’s = 0.344; *F*(16,1096) = 6.45, *p* < .001, *η*^2^ = .086, which appeared to be driven by differences across the (non)religious groups in religious coping, *F*(4,274) = 25.67, *p* < .001, *η*^2^ = .273, emotional-engagement, *F*(4,274) = 4.56, *p* = .001, *η*^2^ = .062, and problem-focused coping, *F*(4,274) = 4.44, *p* = .002, *η*^2^ = .061, but not by emotional-avoidance coping, *F*(4,274) = 1.79, *p* = .131, *η*^2^ = .025.

As seen in Table 3, not surprisingly, Christians and religious “Minorities” reported practicing religious coping significantly more than Atheists (*ps* < .001), Agnostics (*ps* < .001), and those who were Spiritual (*p* = .002 and *p* < .001, respectively). Interestingly, religious “Minorities” (*p* < .001) and those who were Spiritual (*p* = .053), reported significantly more emotional-engagement coping compared to Atheists. Finally, Atheists reported significantly *less* problem-focused coping compared to religious “Minorities” (*p* = .001), Christians (*p* = .041), and those who were Spiritual (*p* = .044).

**Table 3:** Coping strategies (Means, SDs) as a function of (non)religious group.

	Atheist	Agnostic	Spiritual	Christian	Religious “Minority”	*F* (4, 274)	*η* ^2^
Coping	(*n* = 68)	(*n* = 45)	(*n* = 53)	(*n* = 69)	(*n* = 44)		
Problem-focused	1.93_a_ (0.86)	2.19_a,b_ (0.71)	2.33_a,b_ (0.69)	2.31_b_ (0.76)	2.50_b_ (0.73)	4.44^***^	.061
Emotional-Engagement	1.19_a_ (0.65)	1.45_a,b_ (0.49)	1.53_b_ (0.61)	1.38_a,b_ (0.71)	1.71_b_ (0.78)	4.56^***^	.062
Emotional-Avoidance	1.74_a_ (0.77)	2.10_a_ (0.63)	1.99_a_ (0.74)	2.01_a_ (0.85)	1.97_a_ (0.87)	1.79	.025
Religious	0.16 _a_ (0.64)	0.16_a_ (0.47)	0.57_a_ (1.03)	1.33_b_ (1.56)	2.00_b_ (1.44)	25.67^***^	.273

^***^p < .001; Means in the same row that do not share subscripts differ at *p* < .05 (with the exception of the difference in emotional-engagement coping between Atheists and those identifying as spiritual; *p* = .053).

### Religious group differences in self-reported health

An ANOVA revealed self-reported *mental* health differences across the (non)religious groups during the COVID-19 pandemic, *F*(4,275) = 5.34, *p* < .001, *η*^2^ = .072. As seen in Table 4, Agnostics self-reported poorer mental health than did Christians (*p* = .002), religious “Minorities” (*p* = .012), and Atheists (*p* = .050). Also, Christians self-reported significantly better mental health than those who were Spiritual (*p* = .032). However, there were no significant differences in self-reported mental health among Christians, Atheists, or religious “Minorities”.

**Table 4:** Mental and physical health (Means, SDs) as a function of (non)religious group.

	Atheist	Agnostic	Spiritual	Christian	Religious “Minority”	*F* (4,275)	*η* ^2^
	(*n* = 69)	(*n* = 45)	(*n* = 53)	(*n* = 69)	(*n* = 44)		
Mental Health	3.26_a_ (0.99)	2.73_b_ (1.09)	2.91_a,b_ (0.92)	3.43_a,c_ (0.95)	3.18_a_ (1.00)	5.34***	.072
Physical Health	3.57_a_ (0.931)	3.31_a_ (0.925)	3.41_a_ (0.649)	3.23_a_ (0.912)	3.14_a_ (0.795)	2.21	.031

^***^p < .001; Means in the same row that do not share subscripts differ at *p* < .05

An ANOVA conducted to assess differences in self-reported *physical* health, revealed a relatively modest non-significant effect, *F*(4,275) = 2.211, *p* = .068, *n*^2^ = .031, across (non)religious groups. Specifically, Atheists reported marginally better physical health compared to religious “Minorities” (*p* = .089). Clearly, the links to physical health were appreciably weaker than those related to self-reported mental health.

### Group differences in religiosity

The CRS included five dimensions (intellect, ideology, public practice, private practice, and religious experience). A MANOVA to assess differences across these five dimensions of religiosity as a function of (non)religious group identification revealed a significant multivariate effect, Pillai’s = 0.614; *F*(20,1096) = 9.93, *p* < .001, *η*^2^ = .153. However, given that the same pattern emerged across all five dimensions (i.e., with Atheists having the lowest levels of religiosity, followed by Agnostics, Spirituals, Christians, and religious “Minorities”), subsequent analyses were conducted using the composite measure of overall religiosity.

To confirm that individuals’ self-reported religiosity reflected their (non)religious group membership as anticipated, an ANOVA assessed overall religiosity across the groups, which yielded a significant effect, *F*(4,275) = 37.83, *p* < .001, *η*^2^ = .355. Predictably, Atheists reported having the lowest levels of overall religiosity (*M* = 1.50, *SD* = 0.58), followed by Agnostics (*M* = 1.69, *SD* = 0.49), Spirituals (*M* = 2.12, *SD* = 0.72), Christians (*M* = 2.68, *SD* = 1.08), and religious “Minorities” (*M* = 3.09, *SD* = 0.99).

### Relations among religiosity, appraisal-coping processes, and health

Correlations among religiosity, stressor appraisals, coping strategies, and health during the COVID-19 pandemic were also assessed. Of particular interest were the relations between religiosity and the remaining variables. (Correlations among the stressor appraisals and coping strategies themselves are presented in S1 Checklist). As seen in Table 5, greater religiosity was significantly associated with an increased tendency to appraise the COVID-19 pandemic as a challenge from which one might learn and grow, but not with the other types of stressor appraisals. Likewise, religiosity was associated with more positive mental (but not physical) health. Mental health, in turn, was associated with appraising the pandemic as less threatening, less central, and less stressful in general, while being positively associated with appraising the pandemic as a challenge or as controllable by oneself or others. The relations between physical health and stressor appraisals followed a similar pattern, in that better physical health was also associated with appraising the pandemic as less threatening, less central, and less stressful in general, while being positively associated with appraising the pandemic as controllable by oneself (Table 5).

**Table 5:** Means, standard deviations, and correlations among religiosity, stressor appraisals, & health.

Variable	*M*	*SD*	2	3	4	5	6	7	8	9	10
1. Religiosity	2.19	0.99	.003	.25**	.07	.08	.03	.07	.10	.14*	-.04
*Stressor Appraisals*											
2. Threat	2.99	0.83	__	.02	.68**	-.22**	-.14**	.45**	.75**	-.26**	-.30**
3. Challenge	2.66	0.78	__	__	.22**	.51**	.39**	.06	.15*	.15*	.09
4. Centrality	3.24	0.91	*__*	*__*	*__*	-.02	-.04	.24**	.62**	-.24**	-.29**
5. Controllable-by- self	3.46	0.84	*__*	*__*	*__*	__	.56**	-.16**	-.13*	.35**	.22**
6. Controllable-by-others	3.14	0.89	*__*	*__*	*__*	__	__	-.22**	-.09	.24**	.08
7. Uncontrollable	2.31	0.89	*__*	*__*	*__*	__	__	__	.34**	.03	-.06
8. Stressfulness	2.99	0.84	*__*	*__*	*__*	__	__	__	__	-.29**	-.22**
*Health*											
9. Mental	3.18	1.00	*__*	*__*	*__*	__	__	__	__	__	.37**
10. Physical	3.35	0.85	*__*	*__*	*__*	__	__	__	__	__	__

^*^p < .05

^**^p < .01

^***^p < .001

Greater religiosity, as shown in Table 6, was also significantly associated with an increased tendency to report using problem-focused, emotional-engagement, and religious coping to deal with the pandemic. Problem-focused and religious coping were, in turn, associated with greater mental health. Interestingly, however, mental and physical health were both associated with a decreased tendency to report emotional-engagement coping.

**Table 6:** Means, standard deviations, and correlations among religiosity, coping strategies, & health.

Variable	*M*	*SD*	2	3	4	5	6	7
1. Religiosity	2.19	0.99	.28**	.23**	.11	.82**	.14*	-.04
*Coping Strategies*								
2. Problem-focused	2.23	0.79	__	.39**	.44**	.22**	.15*	.04
3. Emotional-engagement	1.42	0.68	__	__	.39**	.29**	-.22**	-.24**
4. Emotional-avoidance	1.95	0.79	__	__	__	.07	-.003	-0.8
5. Religious coping	0.81	1.31	__	__	__	__	.13*	-.09
*Health*								
6. Mental	3.18	1.00	__	__	__	__	__	.37**
7. Physical	3.35	0.85	__	__	__	__	__	__

^*^p < .05

^**^p < .01

### The mediating roles of appraisal-coping processes

#### Stressor appraisals

When the potential mediating roles of appraisals were assessed in the relation between religiosity and *mental* health, the direct relation (*c* path; [62]), B = .13 SE = .05, *p* = .02, remained significant (*c*’ path), B = .13, SE = .05, *p =* .01. The 95% CIs for the potential mediated paths through stressor appraisals each included zero.

When the potential mediating roles of appraisals were assessed in the (non-significant) relation between religiosity and *physical* health, the direct relation (*c* path; [62]), B = -.03, SE = .05, *p* = .51, remained non-significant (*c*’ path), B = -.05, SE = .05, *p =* .24, and the 95% CIs for the mediated paths through stressor appraisals each included zero. Thus, stressor appraisals did not play a mediating role in the relations between religiosity and mental or physical health.

#### Coping

**Fig 1:**
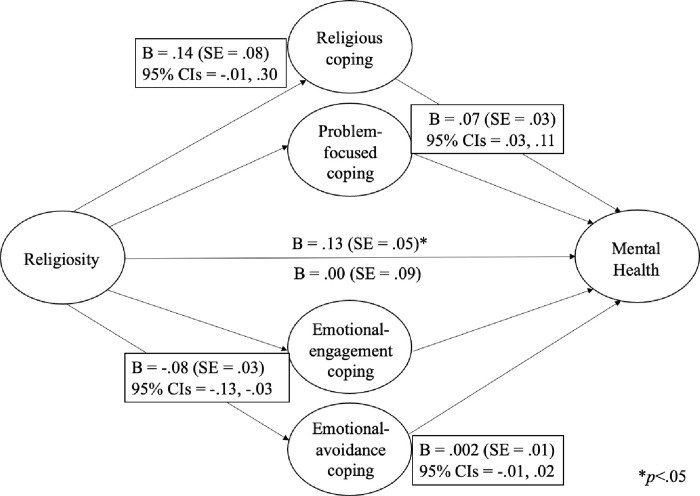
The mediating role of coping in the relation between religiosity and mental health.

When the potential mediating roles of coping strategies were assessed in the relation between religiosity and *mental* health, the direct relation (*c* path; [62]), B = .13, SE = .05, *p* = .02, was reduced to non-significance (*c*’ path), B = -.003, SE = .09, *p =* .97. Moreover, the 95% CIs for the mediated paths through emotional-engagement, B *=* -.08, SE = .03, 95% CI = -.13, -.03, and problem-focused coping, B *=* .07, SE = .02, 95% CI = .03, .11, did not overlap zero (Fig 1).

**Fig 2:**
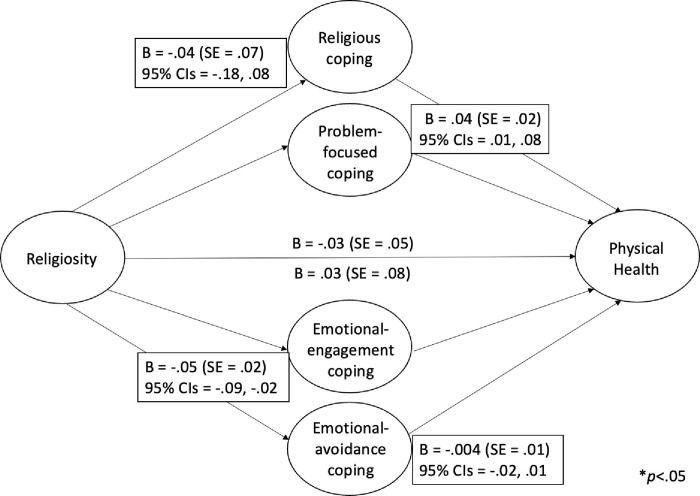
The mediating role of coping in the relation between religiosity and physical health.

When the potential mediating roles of coping strategies were assessed in the (non-significant) relation between religiosity and *physical* health, the direct relation (*c* path; [62]), B = -.03, SE = .05, *p* = .50, remained non-significant (*c*’ path), B = .03, SE = .08, *p =* .76. However, as with mental health, the 95% CIs for the mediated paths through both emotional-engagement (B *=* -.05, SE = .02; CI = -.09, -.02), and problem-focused coping (B *=* .04, SE = .02; CI = .01, .08), did not overlap zero (Fig 2). As a mediation model may involve indirect effects between two variables even in the absence of direct effects [63], emotional-engagement and problem-focused coping appeared to indirectly link religiosity and physical health. Taken together, the use of emotional-engagement and problem-focused coping strategies appeared to fully account for the tendency for more religious individuals to report greater mental health, as well as indirectly link religiosity with greater physical health.

## Discussion

The aim of this research was to examine potential differences in how (non)religious groups appraised and coped with the COVID-19 pandemic, as well as to assess potential relations among religious beliefs, stressor appraisals, coping strategies, and mental and physical health in a religiously-diverse Canadian sample during the pandemic. Given that many countries differed in their approach to dealing with COVID-19, Canada’s pandemic response was also distinct in its implementation on several factors. Some of these included stay-at-home and face-masking requirements, hospital and policy responses, and vaccine rollout, which in turn, affected the severity of COVID-19 case and death counts. At the time of data collection, stay-at-home requirements were still in effect, thousands of people were hospitalized with COVID-19, with over 3000 new cases being reported each day [55]. Thus, the COVID-19 pandemic was a unique circumstance in which to examine the role of (non)religious group identity on stressor appraisals, coping, and mental health within a Canadian population. Several variations emerged across the (non)religious groups in stressor appraisals, coping strategies, and health. While these differences did not always delineate a clear pattern, Atheists predictably reported the lowest levels of religiosity, followed by Agnostics, participants who identified as “Spiritual but not religious”, Christians, and religious “Minorities” reported the highest levels. Religiosity essentially appeared to serve as a proxy for increasing engagement with one’s religious group and belief system (or lack thereof). Moreover, when relations among religiosity, appraisal-coping processes, and health, as well as the potential mediating roles of appraisals and coping were examined, our findings are consistent with the suggestion that greater religious beliefs were tied to better well-being (see [64]) both directly and indirectly through (non-religious) emotional-engagement and problem-focused coping strategies during the pandemic.

### The role of religiosity and stressor appraisals in pandemic-related health

It is commonly accepted that the impact of stressful events are typically related to their controllability, predictability, uncertainty, and chronicity [65]. These characteristics are consistent with the nature of the COVID-19 pandemic as an uncontrollable, unpredictable, and chronic event, including the ongoing threat of the virus itself as well as shifting lockdowns and public health restrictions. Though not in a pandemic context, previous research has demonstrated that greater religiosity and positive religious coping were associated with greater use of challenge appraisals [48], which typically reflect an individuals’ perception that their abilities and resources are sufficient to cope with the danger of the stressor [66]. The present findings within the COVID-19 context revealed that greater religiosity was significantly associated with an increased tendency to appraise the pandemic as a challenge from which one might learn and grow. Earlier research had provided evidence that challenge appraisals are strongly associated with more positive emotional reactions and greater use of cognitive strategies, such as engaging in positive self-statements and active coping [67]. Furthermore, consistent with previous research [48, 67], our findings revealed that an increased tendency to appraise the pandemic as a challenge was associated with more positive mental health (although appraisals did not statistically mediate this relation). It is possible that people greater in religiosity may have been more likely to appraise the pandemic as a challenge given that many religions promote the belief that there is purpose in difficult circumstances because a transcendent force is in control of such situations [68]. Of course, within the religious “Minorities” group, some individuals identified as belonging to a religion that upholds belief in a singular God (i.e., Baháʼí, Jewish, Muslim, or Sikh) which may influence differences in appraising the pandemic as a challenge, compared to those who identified with a religion in which there are multiple gods (i.e., Hindu) or no gods at all (i.e., Buddhist). Nonetheless, religious “Minorities” and Christians were significantly more likely to view the pandemic as a challenge when compared to Atheists. While we found no evidence of significant associations between religiosity and other types of stressor appraisals (e.g., threat, centrality), these results support the notion that religious individuals may have additional capacity to view stressors—including the COVID-19 pandemic—as a challenge rather than as a threat.

### The role of religiosity and coping in pandemic-related health

Not surprisingly, individuals with greater religiosity (i.e., Christians and religious “Minorities”) were significantly more likely to report the use of religious coping. Religious coping was, in turn, associated with greater mental (but not physical) health, in line with previous research [12, 15, 26, 28, 68, 69]. However, religious individuals are not restricted in the coping methods used and were also more likely to endorse the use of (non-religious) problem-focused and emotional-engagement strategies in an effort to deal with pandemic stress. Moreover, both these coping strategies served a mediating role in linking religiosity with greater mental and (indirectly) physical health.

Problem-focused coping—typically comprising strategies such as problem-solving, active coping, and cognitive restructuring—has commonly been linked to higher levels of health and well-being [70, 71]. This profile was similarly apparent during the COVID-19 pandemic in that individuals who coped with pandemic-related stress using problem-focused strategies were more likely to maintain positive well-being [71]. The same was evident in the current study, with a positive relation emerging between problem-focused coping and mental health. As previously reported [72, 73], the current study demonstrated that individuals with greater religiosity also reported an increased tendency to use problem-focused coping to deal with the pandemic (with Atheists reporting significantly lower levels of this coping strategy compared to all other groups with the exception of Agnostics). The tendency for religious individuals to engage in more problem-focused coping is also consistent with earlier research that has suggested links between problem-focused coping and challenge appraisals or social support [74]. Many religious organizations made concerted efforts to foster resilience and maintain social links with their members online throughout the pandemic, even in the absence of physical proximity [75]. These virtual communities may have served as a source of instrumental or informational support [76] for some religious individuals in the context of the pandemic, potentially encouraging more adaptive ways of thinking about and coping with the pandemic, which, in turn, accounted for their more positive mental and (indirectly) physical health.

In addition to problem-focused coping, religiosity was associated with greater use of emotional-engagement coping in the present study, which can include strategies such as emotional expression and emotional social support-seeking [77, 78]. According to our analyses of group differences, this relation appeared to be driven largely by those who identified as belonging to a religious group that is considered a “Minority” in Canada (i.e., Baháʼí, Buddhist, Hindu, Jewish, Muslim, and Sikh). Although emotional-engagement styles of coping have previously been associated with higher levels of well-being [69, 73, 77, 78] our study revealed that emotional-engagement coping was instead associated with more negative mental and physical health outcomes. Although our emotional-engagement factor included strategies such as emotional-expression and social support-seeking, which could have beneficial actions [54, 77, 78], it also included rumination, other-blame, and wishful thinking—strategies that are generally considered to be less “adaptive” in coping with stress [79, 80]. In fact, rumination itself has been associated with diverse psychological disturbances and predicted the later development of depression [81]. In this way, our findings are consistent with recent research in which people with greater religiosity were more likely to “emotionally overreact” and engage in negative coping behaviours during the COVID-19 pandemic [82].

While some religious individuals may have engaged in more positive emotional-engagement coping strategies, such as seeking social support, others may have relied on less constructive emotional strategies to deal with the pandemic. Indeed, greater religiosity has been associated with some unreasonable behavioural coping responses to deal with the COVID-19 pandemic (e.g., hoarding toilet paper), which was also linked with increased emotionality [82]. Moreover, the religious “Minorities” group included participants who identified as belonging to diverse religions, thus differences in religious beliefs as well as differences in the interpretations and practice of those beliefs (e.g., communal worship), may influence the use of more positive or negative emotional-engagement coping strategies. For example, individuals who identified as belonging to religions that observe daily and weekly times of worship (i.e., Jewish and Muslim), may be more likely to benefit from consistent social support-seeking compared to those who identified as belonging to religions that observe less frequent and structured times of communal worship (i.e., Baháʼí, Buddhist, Hindu, and Sikh). Indeed, greater religious participation has long been associated with increased social support [83–85]. Nonetheless, despite higher levels of religiosity being accompanied by greater emotional-engagement coping, which was in turn associated with more negative mental and physical health outcomes, religiosity was significantly associated with more positive mental health. Thus, rather than acting as a direct mediator, emotional-engagement coping appeared to have a suppressor effect [63, 86] in the (positive) relation between religiosity and mental health, such that this relation might have been even stronger (and the relation between religiosity and physical health might have been significant) had it not been for the emotion-focused coping in which religious individuals were more likely to engage.

### Implications for culturally-sensitive pandemic-related health promotion

In addition to illuminating links between religiosity and appraisal-coping processes (including but not limited to religious coping) at the individual level, this research may have implications for public health initiatives that aim to encourage culturally-diverse [87] self-care recommendations and positive mental health practices during times of threat and uncertainty, such as the COVID-19 pandemic or other health crises. Among religious individuals, the positive associations among religiosity, religious coping, and mental health outcomes are consistent with the view that these beliefs may be tied to the endorsement of problem-focused strategies with integrated religious or spiritual elements. Although the belief common to many religious individuals is that seemingly random events can hold purpose or meaning [33, 67] and are in control by a more powerful transcendent force [88], the benefits of religiosity may also come from the adoption of specific appraisals of stressors (e.g., viewing events as challenges rather than threats) and endorsing effective problem-focused coping methods. In the same vein, cognitive restructuring (i.e., reframing one’s interpretation of a situation) may include an element of religion or spirituality, such as prayer and/or mediation, thereby harnessing the benefits typically inherent to both active problem-focused and religious coping together.

Despite the use of problem-focused coping, the present study also revealed the tendency for religious individuals to use emotional-engagement coping, which may be associated with more negative health outcomes. Thus, it may also be prudent for public health messages to caution against the use of certain emotional-engagement coping strategies, such as rumination, denial, or disengagement. While such messaging would be relevant for both religious and non-religious individuals alike, it may be especially pertinent for religious communities in which it would be advantageous to help steer them away from negative religious coping strategies (e.g., anger at God/gods), which have been associated with more negative health outcomes [89]. As religious individuals tend to have formal support networks through their religious organizations and/or communities [75], promotion of this unique resource may help them to cope in more effective ways with both emotional and tangible outcomes.

Non-religious individuals who lack religious communities may likewise benefit from encouragement to engage in social support-seeking and emotional expression with other positive communities in which they may be involved [9]. Indeed, even without considering religious identity, greater social connectedness during pandemic lockdown periods was associated with lower levels of perceived stress, worry, burnout, and fatigue [90, 91]. Moreover, as better mental health was associated with the use of challenge appraisals and problem-focused coping when our study sample was examined as a whole (i.e., including both religious and non-religious individuals), the benefits of engaging in such strategies likely extend within and beyond religious communities. Indeed, given the broad distress created by the COVID-19 pandemic, it has been noted that the pandemic may be thought of as a collective trauma [92–94]. As such, appraising the pandemic as a challenge (rather than a threat) may be associated with post-traumatic growth (i.e., significant positive change arising from major life struggles; [95–97]). Given the potentially long-standing mental health implications of the pandemic, it is important that accessible, tailored, and culturally-sensitive public health initiatives adequately address the needs of people with diverse backgrounds and beliefs.

### Limitations

Like most research, this study has several limitations. First, the religious “Minority” group comprised members from several different religions due to the relatively small number of respondents who identified as belonging to one of a variety of religious groups considered to be “Minorities” in Canada. Thus, there may well be important differences (e.g., individualistic vs. collectivist attitudes or practices; [87]) between these religious groups that were not detected in the current study. Nonetheless, our inclusion of these broad and often under-represented religious groups extends previous research that has in many cases considered Christians alone. Indeed, despite having the least representation within our sample (15.7%), religious minorities were nonetheless over-represented compared to the national average of these groups combined (10.4%; [98]). Likewise, Atheists, Agnostics, and “Spirituals” were over-represented in the current study, perhaps due to the use of MTurk as a data collection tool (which often elicits younger, more highly-educated participants; [99]), thereby offering a valuable opportunity with our screening and validity checks in place; [100] to compare responses across these growing (non)religious groups. Relatedly, as a result of the sample sizes across (non)religious groups, we were unable to adequately assess interactions with gender. Given previous research suggesting that women tend to report poorer mental health (e.g., [101]) and greater religiosity (e.g., [102]) compared to men, as with most research, future studies would benefit from a larger sample of participants. Nonetheless, our patterns of findings held constant even when gender (along with age, race, education, and income) was included as a covariate.

Additionally, mental and physical health were each measured using a single, self-reported item (notwithstanding the documented predictive value of these single-item measures; [103]). Likewise, (non)religious group identity and affiliation was based on a single question as part of the demographic questionnaire, but without a related measure to evaluate the strength of this non(religious) identity. Despite this, our study did include a more precise measure of religiosity, which seemed to capture the relative strength of the identity across the groups (perhaps with the exception of highly-identified Atheists; [104]). A final limitation of this study is the inability to compare our results to those from a pre-pandemic context, which may have provided insight into specific changes in the relations among religiosity, stressor appraisals, coping, and health outcomes that may have occurred as a result of the pandemic. Although our study yielded potentially important patterns among religiosity, appraisal-coping processes and health, given the correlational nature of the data, causal relationships cannot be definitively determined.

## Conclusions

The results of the present study revealed numerous differences in appraisal-coping methods and health across (non)religious groups. However, the clearest patterns emerged between religiosity and health, where both problem-focused and emotional-engagement coping mediated these relations. These findings suggest that greater religious belief may contribute to better mental and physical health, at least in part, through using adaptive (non-religious) coping strategies. Accordingly, this research may have implications for coping at the individual level, as well as informing culturally-sensitive public health messages promoting targeted self-care recommendations with integrated religious or spiritual elements during times of threat and uncertainty, such as the COVID-19 pandemic or future health crises.

## Supporting information

S1 Checklist STROBE statement—checklist of items that should be included in reports of observational studies. (DOCX)
